# Morphologic study of patent ductus arteriosus based on computed tomography data in 25 dogs

**DOI:** 10.3389/fvets.2024.1496944

**Published:** 2024-12-23

**Authors:** Heesung Umh, Kyoung-a Youp, Jeongmin Lee, Daeyun Seo, Seongsoo Lim, Beomkwan Namgoong, Ahreum Choe, Hyeajeong Hong, Nanju Lee, Isong Kim, Junghee Yoon, Jihye Choi, Kichang Lee, Hakyoung Yoon, Min Su Kim

**Affiliations:** ^1^Department of Veterinary Clinical Science, College of Veterinary Medicine and Research Institute for Veterinary Science, Seoul National University, Seoul, Republic of Korea; ^2^Korea Animal Medical Center, Cheongju-si, Chungcheongbuk-do, Republic of Korea; ^3^College of Veterinary Medicine, Jeonbuk National University, Jeonju, Republic of Korea

**Keywords:** patent ductus arteriosus, intervention, computed tomography, morphology, dimension

## Abstract

**Introduction:**

The objective of this study is to analyze the morphology and measurement dimensions of patent ductus arteriosus (PDA) based on computed tomography images.

**Methods:**

The present study retrospectively evaluated computed tomography angiography data from 25 client-owned dogs diagnosed with PDA. PDA was reconstructed based on the central axis and the minimum diameter, ampulla diameter, angle, ampulla cross-section area, and length values were measured at specific measurement sites. Additionally, the minimum diameter ratio, ampulla diameter ratio, ampulla cross-section diameter, and ampulla cross-section diameter/ampulla diameter ratio values were calculated based on direct measurement values.

**Results and discussion:**

The morphology of PDA was distributed as follows: 48% Type IIA, 20% Type IIB, and 32% Type III. A significant correlation was observed between the minimal diameter sagittal and transverse and the ampulla diameter sagittal and transverse, body weight, and angle (descending aorta to PDA). A significant association was observed between ampulla diameter (in both the sagittal and transverse planes) and body weight. The minimal diameter ratio did not demonstrate a significant correlation with the ampulla diameter, body weight, angle and length. However, the ampulla diameter ratio exhibited a significant correlation with the length of the PDA and the angle (descending aorta to PDA). The minimal diameter ratio displayed results that were more closely approximated by a circle, whereas the ampulla diameter ratio showed results that were relatively oval. The ampulla cross-section diameter values differed by an average of 14% from the previously used reference length, ampulla diameter sagittal.

**Conclusion:**

The computed tomography image demonstrated the distinctive cross-sectional configuration of the PDA, which could potentially facilitate advanced pre-procedural planning or the creation of novel occluding devices in the future.

## 1 Introduction

Patent ductus arteriosus (PDA) is common cause of canine congenital heart disease (1). The mortality rate of PDA is reported to be 64% within one year without proper closure and is 16.9 times higher than in patients who undergo closure. Therefore, PDA ductal closure is recommended as soon as possible when left-to-right PDA is diagnosed (2–4). In veterinary medicine, mechanical closure, such as open surgical ligation or minimally invasive techniques, is the mainstay of treatment to close PDAs (4, 5). Recent studies have demonstrated that minimally invasive techniques can be a good alternative for patients with PDA when surgical ligation is not suitable, showing good results with fewer complications and comparable survival rates to surgical ligation (6, 7). However, the placement of inappropriately sized occlusion devices can lead to several sizing complications (8), including device embolization, mild to severe residual flow, and deformation or protrusion of the device (8–16). Although not all of these sizing complications are fatal, severe ischemia, hemolysis and death secondary to embolization has been reported in human (17). Furthermore, embolization-related death has been reported in a dog (18). Therefore, accurate measurement of PDA dimensions and morphology classification (such as minimal duct diameter, ampulla duct diameter, and morphology type) are essential for selecting optimal size of occlusion device (19–22).

Recently in veterinary medicine, trans-arterial angiography with fluoroscopy, transthoracic echocardiography (TTE), transesophageal echocardiography (TEE), and computed tomography (CT) have been used for measuring PDA dimensions (22–28). Angiography with fluoroscopy is commonly used tool and serves as a standard for new measurement methods (20, 25, 28). Fluoroscopy can be used pre-, during, and post-the procedure and can be used to measure dimensions, morphology, and is useful after the procedure to assess proper closure. However, it has limitations of underestimation due to assessing only the sagittal mono plane (22). TTE has the advantages of non-invasive nature, assessment of hemodynamic status, and pre-, intra-, and post-procedural evaluation. However, limitations have been reported, including a tendency for overestimation and limited visualization because of lung interference (20). Transesophageal echocardiography offers advantages over fluoroscopy and TTE, including lung independence, usability throughout the procedure, and minimal measurement deviation. However, it has limitations such as high probe costs, the need for skilled operators, and risks of displacing structures or obstructing PDA and aortic flow (26, 28). The idea of using CT for measurement was proposed to overcome the above limitations. (1) Less prone to overestimation and underestimation that can occur with angiography with fluoroscopy and TTE, (2) visualization of extra cardiac structures is clear because imaging is not limited by lungs (27). In addition, (3) the CT does not cause displacement of structures, which is limitation of TEE, and in human medicine, (4) CT provide more accurate measurements of PDA compared to transthoracic echocardiography. However, CT also has limitations such as radiation hazard, contrast hypersensitivity and repeated anesthesia (29). However, only a few case reports or studies with small numbers of patients have been reported on the measurement of PDA by CT in veterinary medicine. The aim of this study is to present the dimensional features of PDA identified on CT in dogs.

## 2 Materials and methods

### 2.1 Computed tomography data

A retrospective analysis was conducted based on data from client-owned dogs diagnosed with PDA at three different animal hospitals (Seoul National University, Jeonbuk National University, and Chengju Korea Animal Medical Center) from 2016 to 2024. All included dogs underwent thoracic CT scans and angiography as part of the treatment planning process. 25 dogs with well-defined PDA structures were included in this study, whereas 4 dogs with indistinct boundaries with surrounding structures were excluded. The patients underwent CT (Aquillion 64 ™, Toshiba Medical Systems, Tochigi, Japan /ALEXION 16 ™, Toshiba Medical Systems, Tochigi, Japan/Somatom Scope 16 ™, Siemens AG Medical Solutions, Forchheim, Germany) imaging in a head first prone position under endotracheal intubation and general anesthesia. Iodinated contrast agent (Omnipaque ™ 300, GE Healthcare, Oslo, Norway) was administered for angiography. CT data were collected to visualize the PDA, with respiration controlled during the imaging process to minimize movement of the thoracic cavity and internal organs. Measurements of the PDA were taken using the multiplanar reconstruction mode within Radiant DICOM Viewer (64-bit, version 2024.1; Medixant, Poznan, Poland). The measurements were obtained using the length, angle, and closed polygon measuring tools available within the multiplanar reconstruction mode.

### 2.2 PDA reconstruction

**Figure 1:**
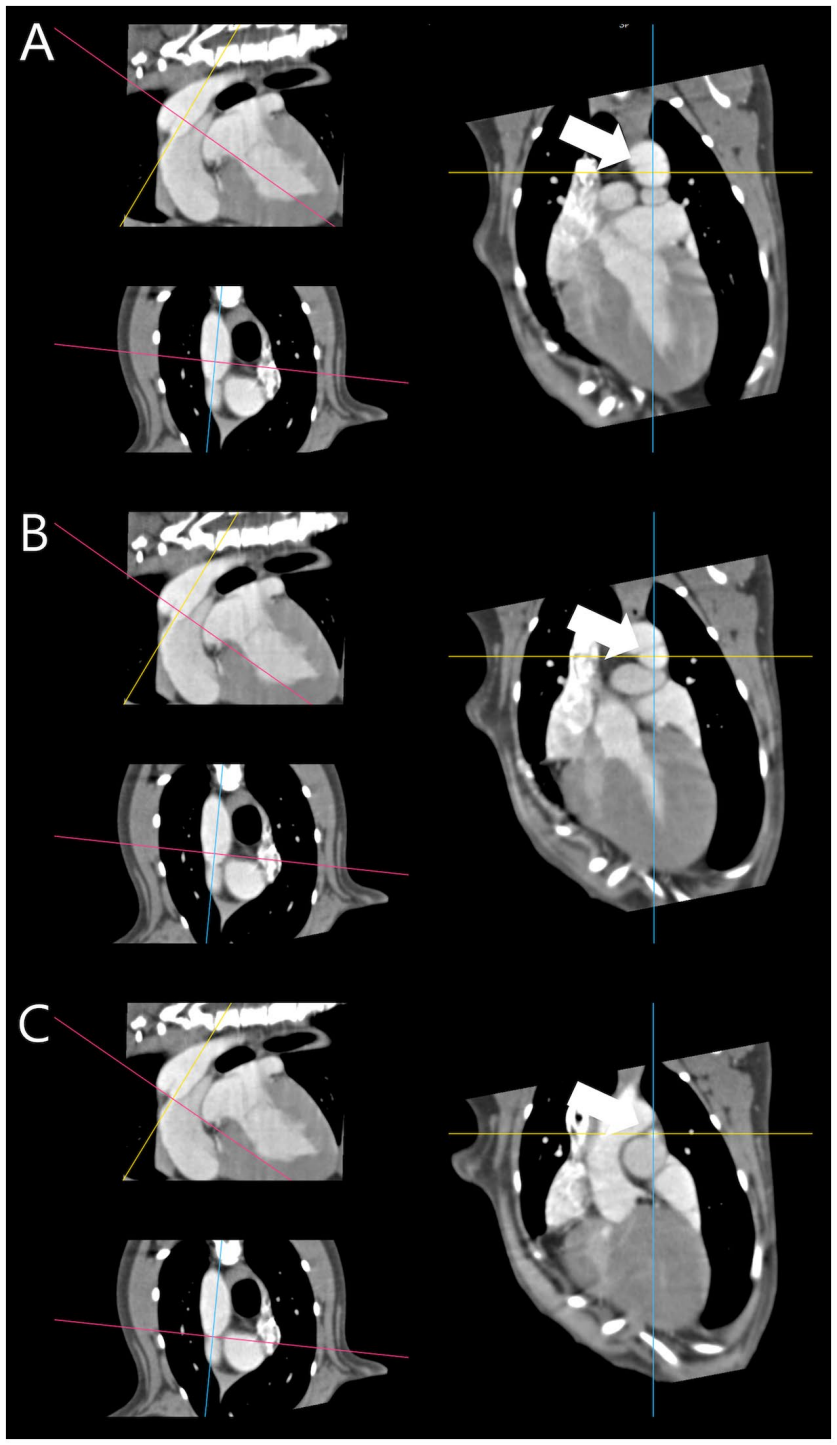
Reconstructed patent ductus arteriosus based on central axis. **(A)** Proximal point centroid (white arrow). **(B)** Mid point centroid (white arrow). **(C)** Distal point centroid (white arrow). The centroids [**(A–C)** – white arrow] coincided on ventral to dorsal scroll without movement of the crossing cursor.

For PDA measurement in CT images, the central axis (30) was defined as follows: The axis along which the centroid of the pulmonary ostium, the distal point of the PDA on the dorsal plane of the CT image, and the centroid of the descending aorta ventral, the proximal point of the PDA, are connected. To measure the vertical cross-section along the central axis, a reconstruction was performed in which the anatomical longitudinal and central axis of the PDA were aligned. After reconstruction, the adequacy of the reconstruction was determined by checking whether the centroids coincided on ventral to dorsal scroll without movement of the crossing cursor on the CT dorsal plane (Figure 1).

### 2.3 PDA morphology classification and dimension measurement

**Figure 2:**
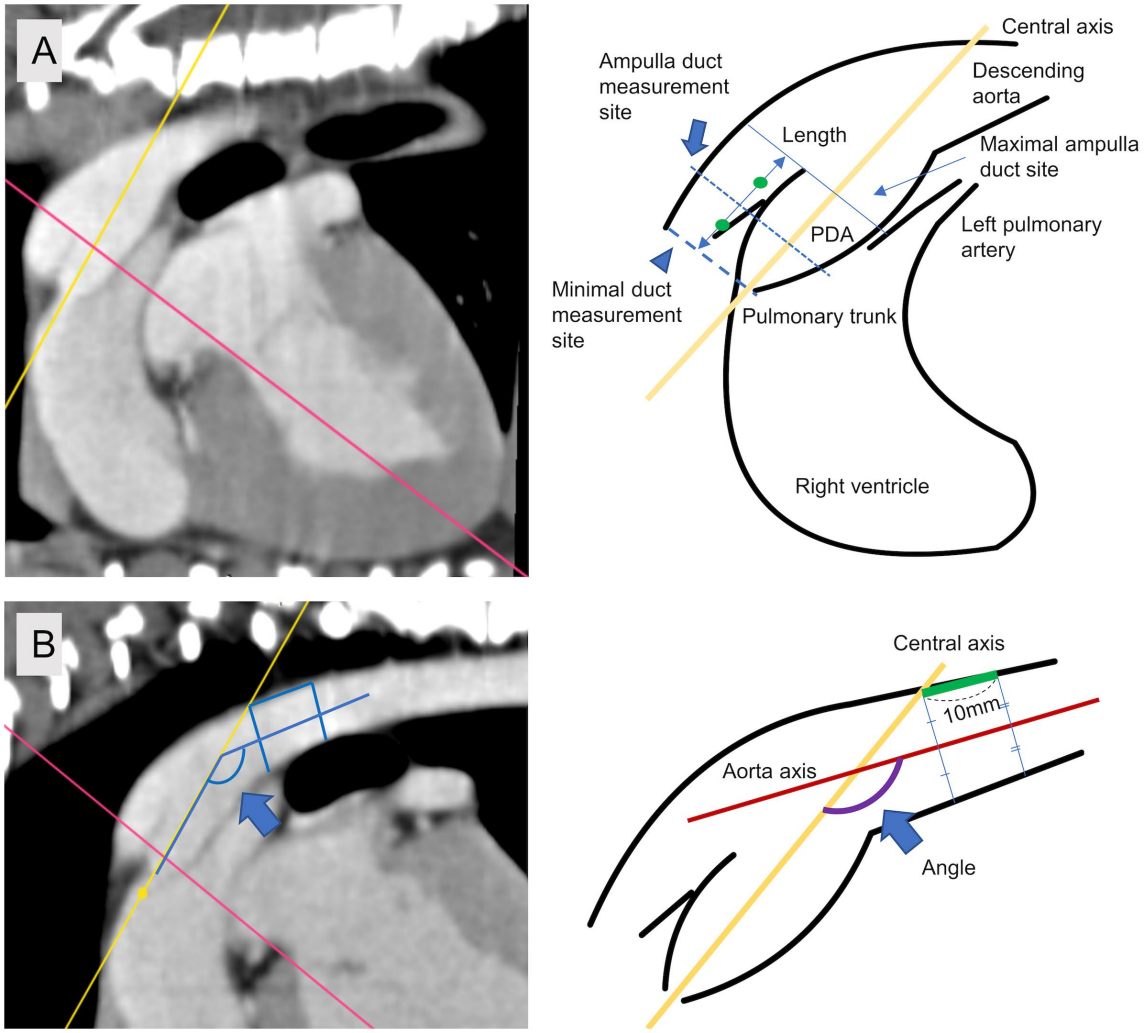
Specific measurement site. **(A)** Minimal duct site (arrow head), ampulla duct site (arrow), length. **(B)** Measuring site of angle between central axis and aorta axis (blue arrow).

**Figure 3:**
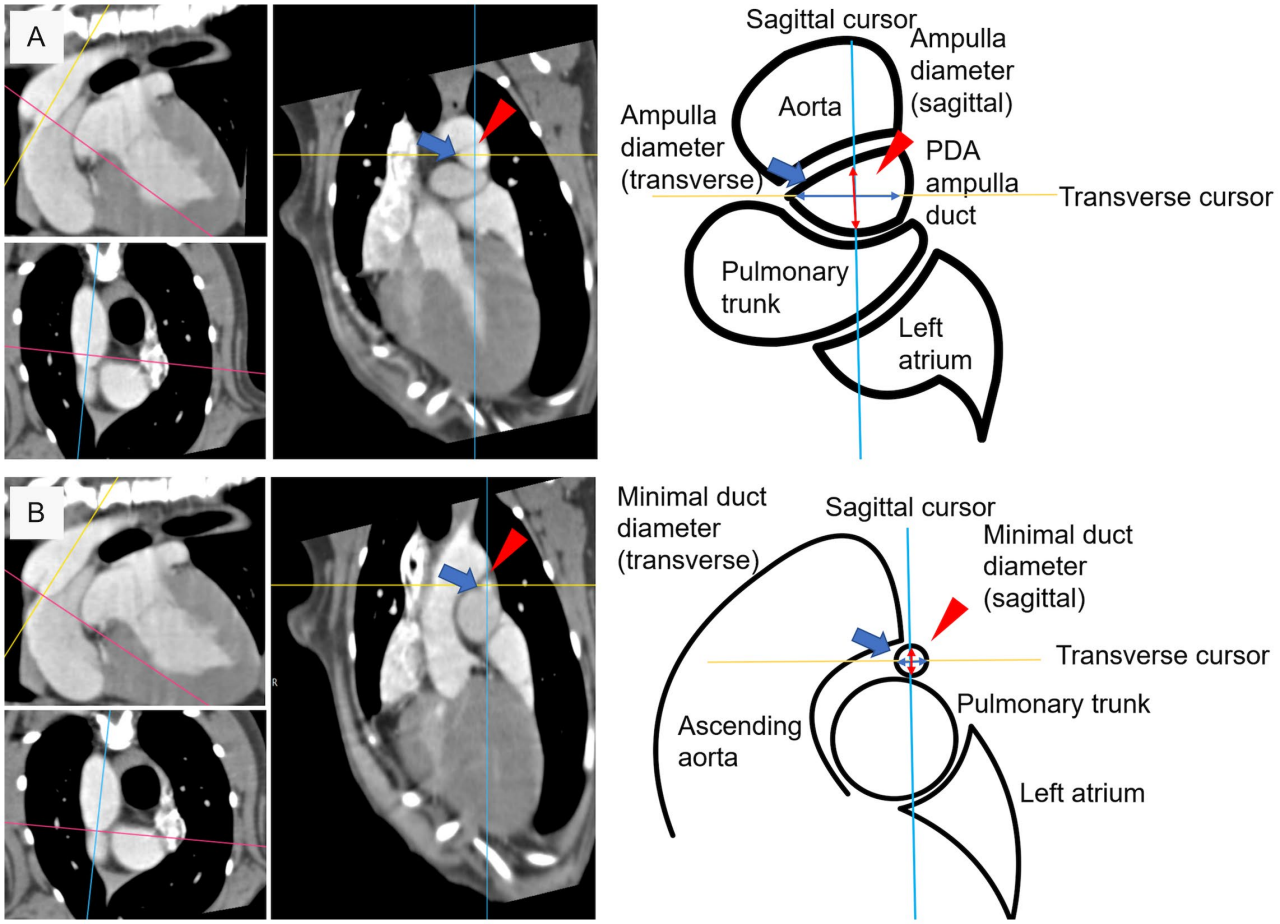
Diameter measuring criteria at specific measurement site. **(A)** Sagittal diameter (red arrow head) and transverse diameter (blue arrow) at ampulla duct site. **(B)** Sagittal diameter (red arrow head) and transverse diameter (blue arrow) at minimal duct site.

The specific measurement site was set as the location where the reference position of fluoroscopy angiography (23) corresponded to the sagittal plane of the reconstructed PDA CT (Figure 2). First, we measured length of PDA from the pulmonary ostium to the junction of the descending aorta and PDA along the cranial border. The minimal duct diameter measurement site was defined as the area with the smallest diameter in the vertical cross-section along the central axis if a clear constriction was identified at the level of the pulmonary ostium, or the area with the smallest diameter in the vertical cross-section along the central axis if no clear constriction was identified at the level of the pulmonary ostium. The ampulla duct diameter measurement site was defined as the midpoint of the length range (Figure 2) and the maximal ampulla diameter measurement site was defined as the proximal connection point between PDA and descending aorta. Diameter measurements were defined as the length of the transverse and sagittal cursors crossing the contrasting structures in cross-section identified in the dorsal plane at specific measurement sites (minimal duct site, ampulla duct site) of the PDA structures as transverse diameter and sagittal diameter, respectively (Figure 3). To measure the angle, we defined the axis connecting the point where the central axis of the PDA intersects the dorsal wall of the descending aorta and the centroid of the segment 10 mm posterior to that point as the aorta axis, and evaluated the degree between the central axis of PDA and the aorta axis as the angle. The morphology classification of PDA was established using angiographic morphology type classification criteria (degree of ductal tapering, and the presence, absence, or location of abrupt ductal narrowing) in dogs (24) and updated morphology type refined through TEE (28), matching the mid-sagittal plane morphology on CT scans. The degree of ductal tapering was based on the calculation of the ratio between the minimum diameter and the ampulla diameter or the maximum ampulla diameter if either the sagittal or transverse diameter met the criteria.

### 2.4 Calculation value

A recent study evaluating the PDA based on TEE (28) reported that the vertical cross-section of the PDA is not perfectly circular. In addition, a human study confirmed that the PDA is a flexible structure that can be deformed by the deployed device, rather than a fixed structure (31). Considering these studies, we quantified the degree of non-circularity in cross-sections at different measurement sites and evaluated calculation values to establish the size relationship between the non-circular PDA cross-sections and the circular occluder device. Based on the directly measured values, Minimal duct diameter ratio (MDR) and Ampulla diameter ratio (ADR) were calculated by calculating the ratio between sagittal diameter and transverse diameter to quantify the degree of non-circularity of the structure. Ampulla cross section diameter (ACD), which calculates the diameter value when the value of ampulla cross section area (Aarea), which is a non-circular structure, is converted to a circle with the same width, was calculated as follow (ACD = 2* $\sqrt{\frac{\mathrm{Aarea}}{\pi }}$ ). To compare the ACD value to the Ampulla diameter sagittal (ADS) value that we had previously used to define the Ampulla diameter on the monoplane, we calculated the ACD/ADS ratio which is the ratio of the difference value ADS to the ACD value.

### 2.5 Statistical analysis

Statistical evaluation was performed using SPSS for windows 29.0.1.0 (IBM) and GraphPad Prism 8 (Dotmatics). Normality was assessed using Shapiro–Wilk test. For comparability with subsequent studies, regardless of normality, we report mean ± SD, median, and range values in Table 1. Pearson correlation coefficient was used to evaluate the correlation between ampulla diameter sagittal (ADS) and ampulla diameter ratio (ADR) and other data that follow normality, and Spearman correlation coefficient was used to evaluate the correlation between minimal diameter sagittal (MDS), minimal diameter transverse (MDT), minimal diameter ratio (MDR), ampulla diameter transverse (ADT), length of PDA, angle (descending aorta to PDA), body weight (BW) and age that do not follow normality. Angle of PDA, length and ADR displayed as linear regression. A *p* value <0.05 was considered to be statistically significant.

**Table 1:** Patient and patent ductus arteriosus measurement data.

	Mean	Median	SD	Range	Normality
BW (kg)	3.09	3.23	2.18	0.93–9.5	No
Age (month)	16.61	12.00	32.66	2–113	No
MDS (mm)	3.26	3.16	1.87	1.54–8.45	No
MDT (mm)	3.51	3.16	2.14	1.49–9.18	No
MDR	0.93	0.97	0.10	0.69–1.06	No
ADS (mm)	4.78	4.89	1.65	2.24–9.47	Yes
ADT (mm)	6.08	5.62	2.44	3.06–13.40	No
ADR	0.79	0.74	0.16	0.57–1.16	Yes
Aarea (cm^2^)	0.23	0.22	0.22	0.06–1.00	No
ACD (cm)	0.54	0.53	0.20	0.27–1.13	No
ACD/ADS ratio	1.14	1.19	0.13	0.88–1.42	Yes
Length (mm)	6.81	7.16	2.80	3.42–15.8	No
Angle (degree)	134.21	137.70	13.36	103.00–156.00	No

Aarea, ampulla cross section area; ACD, ampulla cross section dimeter; ADR, ampulla diameter ratio; ADS, ampulla diameter sagittal; ADT, ampulla diameter transverse; BW, body weight; MDR, minimal diameter ratio; MDS, minimal diameter sagittal; MDT, minimal diameter transverse; PDA, patent ductus arteriosus.

## 3 Results

A total 25 dogs were included in study. The breed distribution of the dogs was as follows: Maltese 32% (8/25), Pomeranian 20% (5/25), mixed breed 12% (3/25), Poodle 8% (2/25), Bichon 8% (2/25), Bedlington terrier 4% (1/25), Cocker spaniel 4% (1/25), Miniature pincher 4% (1/25), Spitz 4% (1/25), Welsh corgis 4% (1/25). The gender distribution consists of females 67% (15, Intact 7, neutered 8) and males 33% (10, Intact 5, neutered 5). The median Age of dogs at the time of CT scan was 12 months (range 2 to 113 months) in 25 dogs. The median Body weight was 3.22 kg (range 0.93 to 9.5 kg) in 22 of the 25 dogs, excluding 3 dogs for which data was lost. In all patients, the course of the PDA is such that the proximal part starts from the ventral part of the aorta and the distal part travels in a cranio-lateral direction before reaching the dorsal part near the bifurcation of the main pulmonary artery. Based on morphology evaluation, the distribution of PDA types was as follows: Type IIA (*n* = 12, 48%), Type IIB (*n* = 5, 20%), and Type III (*n* = 8, 32%), while Type I, Type IV and Type V were not identified in this study. The ductal measurements of PDA and calculation values are presented in Table 1. Minimal diameter sagittal (MDS) was significantly correlated with ADS (Pearson, *r* = 0.839, *p* < 0.001), ADT (Spearman, *r* = 0.542, *p* = 0.005), BW (Spearman, *r* = 0. 546, *p* = 0.009), angle (Spearman, *r* = −0.552, *p* = 0.004), and MDT was significantly correlated with ADS (Pearson, *r* = 0.808, *p* < 0.001), ADT (Spearman, *r* = 0.542, *p* = 0.005), BW (Spearman, *r* = 0. 546, *p* = 0.009), and angle (Spearman, *r* = −0.482, *p* = 0.015). MDS and MDT were not significantly correlated with other variables such as length of PDA and age.

Ampulla diameter sagittal (ADS) was significantly correlated with MDS, MDT, and BW (Pearson, *r* = 0.636, *p* = 0.001), but not with other such as length of PDA, angle, or age. Similarly, Ampulla diameter transverse (ADT) was significantly correlated with MDS, MDT, and BW (Spearman, *r* = 0.522, *p* = 0.013), but not with other length of PDA, angle, or age. Length of PDA was significantly associated with length (Spearman, *r* = 0.551^,^ *p* = 0.004). Angle was significantly associated with MDS, MDT and length of PDA.

**Figure 4:**
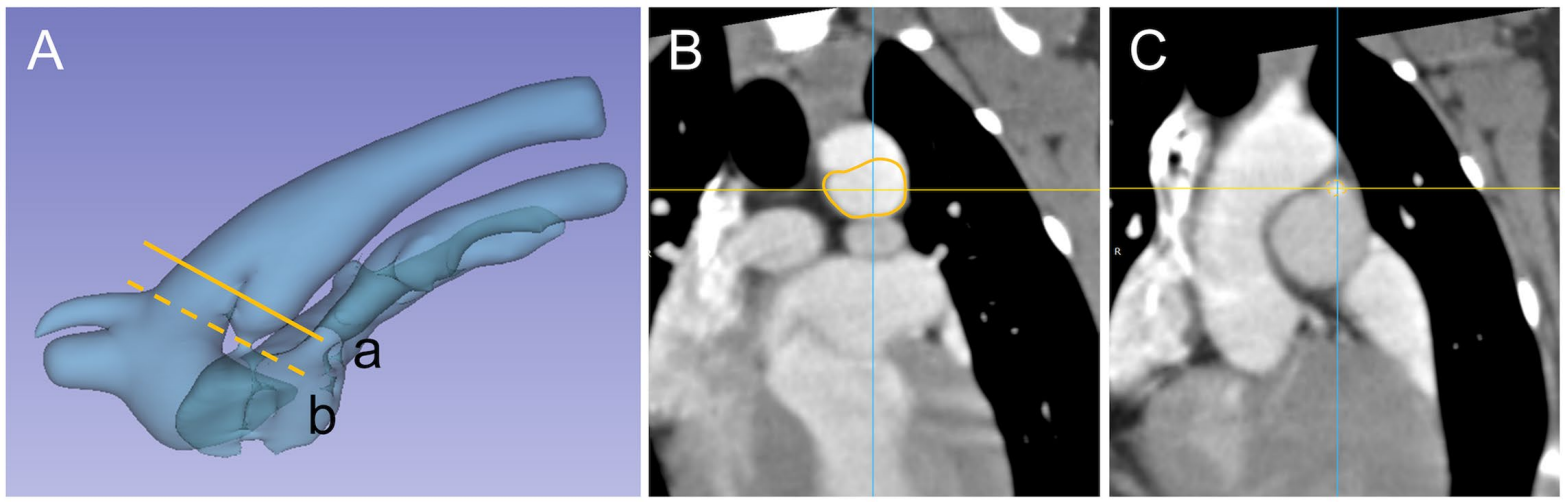
PDA 3D model and cross section appearance at specific measurement site, Patent ductus arteriosus (PDA) and surrounding vasculature displayed as **(A)** a three-dimensional model and specific measurement site (a) ampulla diameter (line), (b) minimal diameter (dash line). Cross section appearance of **(B)** ampulla duct (line) and **(C)** minimal duct (dash line).

MDR did not show a significant correlation with other variables. On the other hands ADR showed significantly correlated with length (Pearson, *r* = −0.467, *p* = 0.019) and angle (Pearson, *r* = −0.677, *p* < 0.001) (Figure 4). The ACD/ADS ratio was significantly correlated with the length (Pearson, *r* = 0.507, *p* = 0.01) and angle (Pearson, *r* = 0.684, *p* < 0.001). No correlations were found with any other variables.

## 4 Discussion

The accurate measurement for device selection during a PDA intervention is of great importance in order to minimize the occurrence of side effects and to ensure the successful closure of the PDA. In order to achieve accurate measurement, this study presented the measurement criteria using CT scan and characterized the measurement dimension. The fact that PDA structure may not be a perfect circle (4), where differences in sagittal and transverse values may exist for minimal diameters, was identified in previous studies (28) and also confirmed in this study. In this regard, the distribution of the shape at the minimum diameter in the population identified in the previous study (28) was found to be oval (17/24, 70.8%) and circular (7/24, 29.2%). However, in this study, the distribution of the shape at the same shape criterion was found to be somewhat different: oval (5/25, 25%) and circular (20/25, 75%). A possible reason for this difference is the difference in the criteria axis used. In this study, the pulmonary ostium or minimal duct was measured at the smallest diameter part of the central axis or the area where the axis connects to the pulmonary artery in previous studies based on dimensional measurements, whereas in studies based on 3D-TEE, a cross section across the pulmonary ostium was first established and then axis perpendicular to it (28). This difference in the slope of the representative axis is considered to be a factor that may have caused the difference in proportions.

Ampulla diameter ratio is similar to the previous study (28) in that the cross section of the ampulla has an oval shape rather than a circular shape, and in this study, it was found to be close to an oval shape with a length difference between ADS and ADT. The difference in the proportion was identified in a previous study (28) as oval shape (19/24, 79%) and circular shape (5/21, 21%), and this study showed a similar distribution of proportions as oval shape (19/25, 82%) and circular shape (6/25, 18%) when evaluated using the same criteria (28) as the previous study. Furthermore, similar to previous study (28), we found that the ratio of MDR to ADR is not always a consistent structure with the same circular and oval shapes. In addition, the significant negative correlation between ADR and length of PDA and angle suggests that PDA with angle and length larger than the representative values are likely to have a more oval shaped cross-sectional area. We consider that this may reduce the possibility of error in device size selection due to overlooking the transverse diameter due to interpretation based on mono-plane evaluation in the conventional sagittal plane diameter. Based on the results of this study and previous studies (28), it can be seen that the measurement plane should be considered when measuring structures that are considered to be non-perfectly circular (Figure 4), as the difference in the measurement value may be due to the characteristics of the structure rather than the difference in the tool. It is therefore necessary to further refine the measurement diameter in order to align it with the measurable dimension structure of the tools, which can be classified into the following categories: Sagittal diameter tool (Trans-arterial angiography, TEE-2D), Transverse diameter tool (TTE-R and TTE-L), Sagittal + transverse diameter tool (TEE-3D, CT) plane.

**Figure 5:**
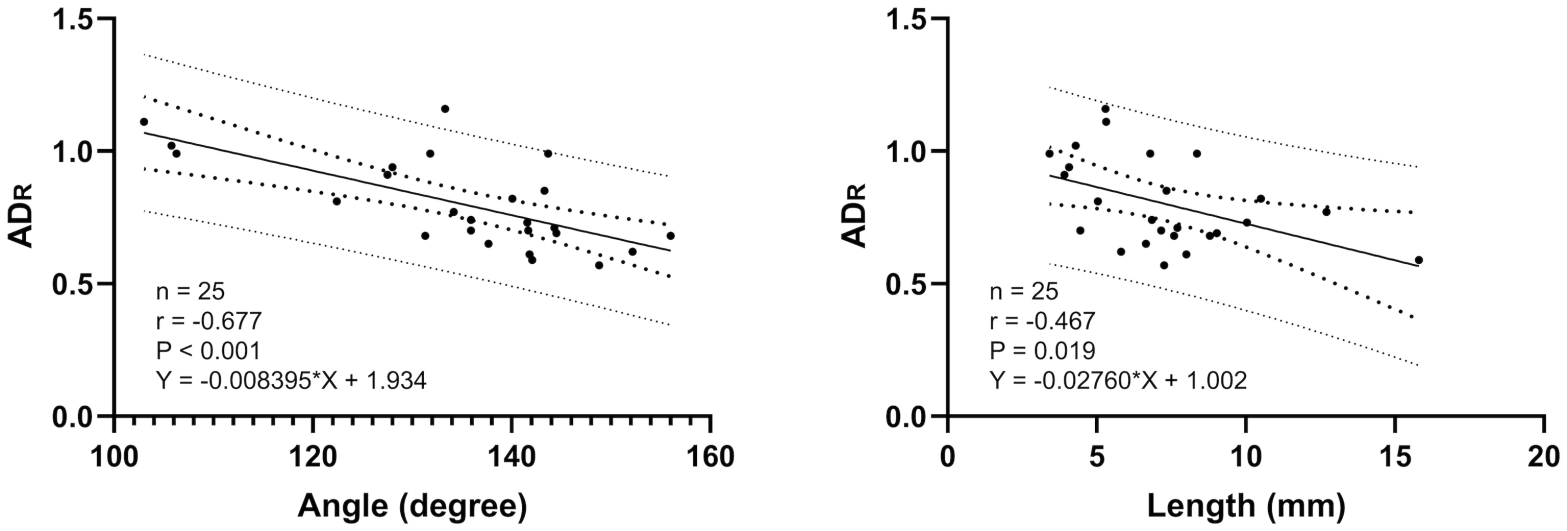
Linear association of PDA length (mm), angle (degree) and ADR. The solid line represents the line of best fit, the inner larger dotted lines represents the 95% confidence intervals for the best fit and the outer dotted lines represents the 95% prediction interval for the observation.

Compared to the mean angle of PDA 148.8 ± 7.6 degrees (range 117 to 164, median 150) in a previous trans-arterial angiography study (27) that presented the angle between the descending aorta and PDA, we showed that the mean angle of PDA 134.2 ± 13.63 degrees (range 103 to 156, median 137.7) was smaller in this study. In three dimensions, the PDA is mostly traveling in the cranio-ventral direction, and in this regard, we confirmed that there may be some differences in the angle between the data evaluated in the mono-plane and the multiplane (Figure 5). In the future, it is considered that the newly considered angle may be helpful for the development of catheters aimed at trans-arterial access.

The ACD/ADS ratio, one of the calculations values, was investigated to determine the correlation between the circularly designed occlusion device and the PDA ampulla, which is identified as oval. Currently, most size criteria for occlusion devices are based on a single diameter measured on a mono-plane, and there is no literature that considers the property of non-circular structures that can have multiple diameters depending on the criteria. In addition, as it has been reported in the literature that PDA structures can be flexibly stretched rather than fixed (31). We checked the ratio of the difference between the diameter of the non-circular cross section (ACD) and the ADS, a mono-plane measurement that has been used as the basis for setting the size of the occlusion device. The ACD/ADS ratio was found to be mean 1.14 ± 0.13 (range 0.88 to 1.42, median 1.19) indicating that the ADS value was measured as much as 14% smaller than the ACD value. Considering these differences, we propose to consider replacing the traditional device selection criteria, ADS, with an ACD that is tailored to the cross-sectional width of each patient. As opposed to the previous, it has been confirmed in both human and veterinary studies that deformation of the device may occur due to excessively oversized devices or the structure of the PDA (17, 32). In this regard, it is considered that future research: (1) the shape of PDA that flexibly deforms due to device expansion and (2) the maximum expansion level relative to the new reference diameter that does not cause device deformation may be a good idea to solve the current sizing complication.

This study has several limitations. First, it is unclear whether the minimal duct diameter site set relative to the central axis is the same site as the conventional measurement, which is a direct measurement of the structure across the pulmonary ostium. This is due to the fact that the minimal duct site is a smaller structure and more tortuous structure compared to other measurement dimensions. Second, the statistical reliability is reduced due to the small number of patients. Third, the comparison with fluoroscopy (angle between PDA and descending aorta) was based on statistical data of previous studies, not within the same individual, making it difficult to confirm statistical significance. Fourth, it is a retrospective study and the interpretation is based on CT scans taken under different conditions, which makes the data inconsistent across conditions. Nevertheless, it is valuable to present a measure of PDA based on CT, which can serve as a comparison and measurement standard for future studies. As a further study, it is considered necessary to present a representative value of PDA patients based on CT data from a larger study, and to establish the substitutability and role of tools based on the comparison of multiple tools in the same patient for future intervention procedures.

In conclusion, computed tomography (CT) images are capable of assessing morphology and dimensions in a conventional tool. The distinctive cross-sectional shape, which can only be observed in three-dimensional structures, represents the primary distinction between this approach and conventional measuring tools. It is anticipated that CT will contribute to a reduction in sizing complications in future interventional procedures through the implementation of sophisticated pre-planning techniques or the development of new devices.

## Data Availability

The datasets presented in this study can be found in online repositories. The names of the repository/repositories and accession number(s) can be found in the article/supplementary material.
